# Supplementary material for: Prevalence and determinants of undernutrition among adolescents in India: A protocol for systematic review and meta-analysis
Source: PLoS One. 2022 Jan 24;17(1):e0263032. doi: 10.1371/journal.pone.0263032 (PMC8786165; doi:10.1371/journal.pone.0263032)
Supplement: Attachment — Submitted filename: Review paper PLOS.docx.pdf [file pone.0263032.s004.pdf]

In this manuscript, the authors presented the “Prevalence and Determinants of Undernutrition among Adolescents in India: A Protocol for Systematic Review and Meta-Analysis”. The manuscript has an interesting topic to study through a systematic review and meta-analysis, the proposed study is clear and valid, the hypothesis is good and the methodology is feasible; however, some issues need to be addressed.

General

- Please, revise the English grammar, vocabulary, and punctuation. There are grammatical errors and sometimes it is hard to understand the text.

Abstract

- Methodology: You should include the registration number of the PROSPERO protocol.
- Discussion: in the last sentence you could include the contribution to the creation or improvement of public policies to fight undernutrition.

Introduction

- In the 2nd paragrapher you should provide an explicit statement of the question(s) the review will address with reference to participants, interventions, comparators, and outcomes (PICO) (according to the PRISMA)

Objectives

- You might review the format of the objective.

Methods

Systematic review registration and reporting of the findings

- You should review the PRISMA-P checklist (attached file);
- You have to include the PROSPERO number.

Study design and search strategy

- Did you do a pilot search to define the MeSH terms and search strategies? Have you validated them?
- You should include more keywords as synonyms to adolescents like teenagers or young adults.
- You should organize the MeSH terms into blocks to retrieve the publications and show them.

Inclusion criteria

- "All articles published only in the English language" Why not in Hindi?

Risk of bias and quality assessment

- Why do not submit the studies to a critical appraisal using the Joanna Briggs Institute Critical Appraisal checklist (JBI 2020) and Grading of Recommendations Assessment, Development, and Evaluation (GRADE)?

Data analysis and assessment of publication bias

- How many studies do you need to perform the meta-analysis?
